# A study protocol for a systematic review and meta-analysis on macroscopic body movements as a marker for acute stress

**DOI:** 10.1186/s13643-025-03050-4

**Published:** 2026-01-12

**Authors:** Miriam Kurz, Veronika Ringgold, Luca Abel, Felicitas Burkhardt, Robert Richer, Lena Schindler-Gmelch, Anne I. Turner, Bjoern M. Eskofier, Nicolas Rohleder

**Affiliations:** 1 Chair of Health Psychology, Friedrich-Alexander-Universität Erlangen-Nürnberg, Nägelsbachstraße 49a, 91052 Erlangen, Germany; 2 Machine Learning and Data Analytics Lab, Friedrich-Alexander-Universität Erlangen-Nürnberg, Carl-Thiersch-Straße 2b, 91052 Erlangen, Germany; 3 Chair of Clinical Psychology and Psychotherapy, Friedrich-Alexander-Universität Erlangen-Nürnberg, Nägelsbachstr. 25, 91052 Erlangen, Germany; 4 Institute for Physical Activity and Nutrition, Deakin University, 221 Burwood Highway, Burwood, Victoria 3125 Australia

**Keywords:** Acute stress, Stress assessment, Body movements, Macroscopic movements, Facial expressions

## Abstract

**Background:**

Experiencing stress is a normative part of human life. Due to its major impact on health and longevity, effort has been put into understanding underlying determinants and consequences. For stress measurement, a vast number of different methods, including physiological, subjective, and behavioral assessments, can be used. Previous measurement methods have already provided valuable insights into the role of the stress systems. However, their application has some drawbacks, highlighting the need for more convenient, less expensive, less intrusive, and continuous methods. A promising alternative could be the assessment of macroscopic body movements, which we define as body movements that are actively, consciously, or unconsciously, initiated by the muscular system.

**Methods:**

The study protocol was registered with PROSPERO, and PRISMA-P guidelines were followed. We will screen MEDLINE, PsycInfo, Web of Science, Embase, and Google Scholar for suitable studies in German or English with no restrictions towards the publication date. To do so, an a priori defined search strategy will be employed. Furthermore, we will carry out backward and forward citation searches. Inclusion criteria comprise studies examining macroscopic body movements in response to stress or related psychological constructs induced by standardized protocols. Exclusion criteria include studies on non-human populations as well as studies that do not report on a control group/condition nor pre-/post-comparisons nor traditional stress markers. The search, selection, and data extraction processes will be performed by two reviewers independently, while a third reviewer will be consulted in case of discrepancy. We will assess Risk of Bias using the revised Cochrane Risk of Bias tool or ROBINS-I, as appropriate. We will conduct random-effects meta-analyses where feasible. The quality of evidence will be evaluated using the GRADE approach. Furthermore, results will undergo critical examination towards meta-biases.

**Discussion:**

Due to the growing number of studies assessing macroscopic movements in the context of stress, the field is becoming complex, highlighting the need for a summary of current findings. Thus, here we outline a study protocol for a systematic review and meta-analysis on macroscopic movements as a potential novel marker for stress.

**Systematic review registration:**

PROSPERO CRD42024539659

**Supplementary Information:**

The online version contains supplementary material available at 10.1186/s13643-025-03050-4.

## Background

The primary objective of the human body is to preserve a state of homeostasis, which involves regulating inner processes to achieve a “state of balance” [1]. When facing a stressor, its perception via the central nervous system (CNS) ultimately activates the locus coeruleus–norepinephrine system [2] and the paraventricular nucleus of the hypothalamus [3]. In turn, the two human stress systems, the sympathetic nervous system (SNS) as part of the autonomic nervous system (ANS) and the hypothalamic–pituitary–adrenal axis (HPA axis), are activated. The SNS reacts within seconds and prepares the body for fight-or-flight by releasing adrenaline and noradrenaline. The HPA axis reacts more slowly and releases the stress hormone cortisol, which, in complex orchestration with the ANS, enables the organism to cope with the stressor [4–9]. Short-term stress responses are beneficial and necessary to ensure survival by enabling an adequate response to threatening stimuli [10, 11]. However, long-term stress, and stressors that are recurring and associated with emotional or physical strain, can exert harmful effects on human health [12–14] due to, e.g., dysregulation of the HPA axis and the ANS [15], and increased inflammation [11].

Given the negative impact that particularly long-term stress can exert, it is important to understand its determinants, mitigate its consequences, and provide a basis for designing more effective interventions to buffer the effects of stress and therefore foster well-being. Laboratory stress protocols allow for standardized stress exposure and assessment of the individual’s behavioral, physiological, and self-reported stress responses [16, 17]. Thus, they can help to understand underlying mechanisms of the human stress response and draw causal conclusions [10, 18, 19]. Alongside protocols such as the Socially Evaluated Cold Pressor Test (SE-CPT; [20]) or the Montreal Imaging Stress Task (MIST; [21]), the Trier Social Stress Test (TSST; [22]) is considered the gold standard to induce acute stress in the laboratory [10, 23]. Of these laboratory stress protocols, the stress response can be assessed in various ways. Subjective self-reports, for instance via standardized state questionnaires like the Short Stress State Questionnaire (SSSQ; [24, 25]), Positive Affect Negative Affect Schedule (PANAS; [26]), or the Primary Appraisal Secondary Appraisal (PASA) questionnaire [27], offer a reliable and cost-effective, but time-delayed and non-continuous method to interrogate inner states and thus quantify the subjectively perceived stress [26, 28]. In addition, they, like all self-reports, might suffer from recall bias [29]. Further, biological measures, such as hormone (e.g. cortisol; [30, 31]) or enzyme (e.g. alpha-amylase; [32]) levels in blood, urine, or saliva, provide insights into the (re-)activity of the HPA axis or of the SNS, respectively [33]. Although such hormonal or enzymatic measures offer a dependable and non-invasive means of assessment [34], they are also subject to confounding influences such as hormonal diurnal cycles [35, 36], medication or food intake [37], or mental and physical conditions [38–40]. Further, they do not allow for continuous assessments [28, 41], and are associated with considerable laboratory cost and work [42, 43]. Physiological variables, such as electrocardiogram (ECG), skin conductance, respiration, or electroencephalogram (EEG) data may provide insights into the activity of the SNS [28, 44]. Heart rate (HR) and HR variability (HRV), for instance, have been found to be a reliable marker for stress assessment [45–48]. However, while HR(V) can now be measured in a minimally obtrusive manner using wearable trackers like smartwatches [45, 49], more complex ECG data can so far only be collected by a varying number of electrodes on the upper body [46], which potentially interferes with participants’ natural experience and behavior.

Thus, it can be summarized that while there is a plethora of possibilities to study stress in a standardized fashion, there is a lack of methods allowing for non-invasive, non-intrusive, continuous, and inexpensive assessment of stress. One innovative approach that may bridge this gap is the contactless assessment and interpretation of body movements. Body movements can be classified into macroscopic and microscopic movements. We define macroscopic movements as body movements that are actively initiated by voluntary and/or involuntary activation of skeletal muscles, and microscopic movements as all body movements that are indirectly caused by internal physiological processes (such as the beating of the heart or respiration-induced chest movements). With regard to macroscopic movements, a long-established methodology entails the evaluation of facial expressions [50, 51]. A limited number of studies are available that describe how facial expressions are associated with stress responses. Lupus et al. (2014) report links between the expression of anger and HPA axis and SNS activity following the TSST in men, but not in women [52]. Blasberg et al. (2023) report correlations between movements of different regions of the face, such as the upper eyelid, the upper lip, or the lip corner, so-called Action Units (AUs), with the psychosocial stress response, including cortisol, to the TSST for both women and men [53]. From such AUs, emotion indices may be retrieved [54]. Using Machine Learning algorithms, Giannakakis et al. (2017) have established a pipeline to automatically detect and analyze AUs from video recordings [55].

Cues about people’s inner states may not only be visible in their facial expressions but may also be inferred from their body posture. The concept of “freezing”, which is characterized by a decrease in heart rate as well as a reduction in body sway, has received attention as a marker for social threat [56]. Participants were found to show more freezing behavior in response to stimuli such as angry faces [56] or unpleasant films [57]. While freezing refers to rapid reductions in body movements and HR in the immediate response to a threat, we and others have found that stress responses that develop more slowly are also associated with a reduction of body movements. We found a decrease in head, upper extremities, and upper body movements in response to the TSST [58]. Similarly, Pisanski et al. (2018) reported a decrease in hand movement, following the TSST [59].

Besides the approaches using facial expressions and reduction of macroscopic body movements, a smaller body of research has looked at further possibilities to assess stress and related psychological states. For instance, one study detected variations in pressure distribution on an office chair after a laboratory stress intervention [60]. By assessing the angular movement of a steering wheel, Paredes et al. (2018) reported that this allowed them to distinguish between a stressed and a non-stressed state of participants driving a car [61]. Some studies have explored alterations in keyboard typing patterns before and after stressful events. While some of the results suggest a possibility of differentiating between high and low stress conditions based on keyboard typing behavior [62, 63], others found no universal patterns [64, 65].

Thus, it can be stated that there currently are a number of different approaches aiming to utilize body movements as stress markers. In light of recent technological advances, such as the prediction of human pose through images or videos (see e.g., [66]), this research field can be expected to grow rapidly over the next few years. Hence, it is necessary to provide an overview of the current literature, and, thus, a framework for further research. This systematic review and meta-analysis examine the reliability and validity of macroscopic body movements as a marker of acute stress or related psychological constructs in humans, either by comparing responses under experimentally induced stress to baseline or non-stressful conditions, or by including studies that assessed traditional stress markers alongside macroscopic movements.

## Methods

### Protocol registration and reporting information

This systematic review was registered with PROSPERO CRD42024539659. This protocol, as well as the ensuing systematic review/meta-analysis, is reported in accordance with PRISMA-P guidelines [67] (see Additional file 1).

### Classification of body movements

In our research methodology, we employ a categorization approach to identify suitable studies. Specifically, we distinguish between macroscopic movements and microscopic movements. Macroscopic movements are, for example, body posture and/or its change over time, or behaviors such as keyboard typing. Moreover, macroscopic movements may further be classified into movements that involve an object, like a steering-wheel or a keyboard, and object-free movements, such as body sway. An example of a microscopic movement (which is not part of the planned review) is the upward and downward motion of the rib cage during inhalation and exhalation, which is caused by respiratory activity. Although these chest movements are visible to the human eye and could be classified as either a macroscopic or a microscopic movement, we have explicitly chosen to exclude them from the current review. This decision was made to narrow the scope of the review and to maintain a clear focus on movement patterns primarily driven by motor behavior.

### Eligibility criteria

We will select studies based on the criteria study design, participants, exposure/interventions, and outcomes (PICO/PECO framework; [68]).

- *Study design, comparison, and exposures/interventions*: We will include studies that actively induce acute stress or related psychological states, i.e., perception of threat or negative affect, in human study participants via a standardized protocol (e.g., via laboratory stress protocols such as the TSST or MIST, or via experimental paradigms that systematically manipulate real-life environmental factors such as office noise), and that report a comparison between a stressed group or condition and an appropriate control group or condition in the desired outcome variables. This could be achieved by comparing different experimental groups (between-subject) or conditions (within-subject). We will also include studies reporting only on pre-post comparisons before and after the stressor if their measures include both an established marker of stress (e.g., saliva-based markers such as cortisol, blood-based markers such as adrenaline/noradrenaline, or electrophysiological markers such as HR(V) or skin conductance) and a measure of macroscopic movement. Studies that investigate the opposite relationship between body movement and stress, e.g., the effect of physical exercise on the stress response, will be excluded from the analysis.
- *Participants*: In the systematic review, we will include studies that exclusively involve human participants. Animal studies will be excluded from our analysis. With regards to the human sample, there will be no limitation, i.e., we will include studies reporting outcomes in children, adolescents, and adults as well as studies that focus on either the entire population or specific sub-populations. Furthermore, we will include patient groups independent of the presence/absence of any disease/disorder.
- *Outcomes*: We will include studies that report on at least one macroscopic movement which we classify as any movement that is, consciously or unconsciously, evoked by the muscular system. Studies may also include established indicators of the human stress response. However, if only traditional measures of stress are reported, we will not include the study in this review.

There will be no restrictions based on the publication date. This is due to our anticipation that relevant research is more likely to be recent, and we aim to avoid excluding valuable foundational literature in an already limited pool of literature. However, we will only consider articles published in peer-reviewed journals or peer-reviewed conference proceedings, written in either English or German. Conference abstracts, dissertations, and other gray literature will not be included in the study selection.

### Information sources and search strategy

As a primary source for identifying eligible papers related to our systematic review on stress and macroscopic movements, we will conduct a comprehensive search in the following electronic databases: MEDLINE, PsycInfo, Web of Science, Embase, and Google Scholar. These databases were chosen for their extensive coverage of scientific literature in the fields of psychology, medicine, and related disciplines. We will screen the title and abstract of each paper to determine its eligibility based on a previously developed guideline. Additionally, we will employ a citation chaining technique to identify additional relevant studies. This approach involves reviewing the reference lists of included studies to find other potentially eligible articles that might have been missed in the initial database search. The systematic search will be performed by using combinations of relevant keywords related to stress and macroscopic movements. These keywords were derived based on an exploratory literature scan and refined through team consensus (MK, VR, LA, FH, LSG, RR, AT, and NR), informed by prior relevant publications and iterative testing in different databases. The final search strategies for PsycInfo, MEDLINE Complete, and Embase are displayed in Tables 1, 2, and 3, respectively. For Google Scholar, due to its large volume of search results, we will screen only the first 10 pages of search results (i.e., the top 100 entries), as has been done previously [69]. This step is intended to identify additional potentially relevant studies that may not have been indexed by the other databases. We will use Web of Science for forward and backward citation searches.

**Table 1:** Search string for PsycInfo. The search combines the concept stress with the concept macroscopic movements

PsycInfo search			
		Filter	Search string
Concept 1: Stress		Title/Abstract	("acute stress" OR "psycho* stress" OR "stress respons*" OR "stress react*" OR "stress exposure*" OR "stress detection*" OR "stress measur*" OR “stress intervention*” OR “stress induc*” OR “Trier Social Stress Test” OR TSST OR “social* threat*” OR “threat* social”)
	OR	Subjects/Mesh terms	"Cardiovascular Reactivity" OR "Stress" OR "Acute Stress" OR "Perceived Stress" OR "Physiological Stress" OR "Stress Reactions" OR "Distress" OR "Emotional Exhaustion"
Concept 2: Macroscopic body movements	AND	Title/Abstract	(motion* OR gait OR postur* OR sway OR gesture* OR "Facial Action Coding System" OR "facial expression*" OR “action unit*” OR freezing OR biometrics OR "pressure distribution" OR "capacitive mouse" OR "pressure sensitive")
	OR	Subjects/Mesh terms	"Facial Recognition (Artificial Intelligence)" OR "Sensor Technology"

**Table 2:** Search string for MEDLINE complete. The search combines the concept stress with the concept macroscopic movements

MEDLINE complete search			
Concept 1: Stress		Title	("acute stress" OR "psycho* stress" OR "stress respons*" OR "stress react*" OR "stress exposure*" OR "stress detection*" OR "stress measur*" OR “stress intervention*” OR “stress induc*” OR “Trier Social Stress Test” OR TSST OR “social* threat*” OR “threat* social”)
	OR	Abstract	("acute stress" OR "psycho* stress" OR "stress respons*" OR "stress react*" OR "stress exposure*" OR "stress detection*" OR "stress measur*" OR “stress intervention*” OR “stress induc*” OR “Trier Social Stress Test” OR TSST OR “social* threat*” OR “threat* social”)
	OR	MesH Terms	Stress, Physiological OR Stress, Psychological OR Trier Social Stress Test OR Trier Stress Test
Concept 2: Macroscopic body movements	AND	Title	(motion* OR gait OR postur* OR sway OR gesture* OR "Facial Action Coding System" OR "facial expression*" OR “action unit*” OR freezing OR biometrics OR "pressure distribution" OR "capacitive mouse" OR "pressure sensitive")
	OR	Abstract	(motion* OR gait OR postur* OR sway OR gesture* OR "Facial Action Coding System" OR "facial expression*" OR “action unit*” OR freezing OR biometrics OR "pressure distribution" OR "capacitive mouse" OR "pressure sensitive")
	OR	MesH terms	"Facial Recognition" OR "Automated Facial Recognition" OR "Digital Health"

**Table 3:** Search string for Embase. The search combines the concept stress with the concept macroscopic movements

Embase search			
Concept 1: Stress		Title	("acute stress" OR "psycho* stress" OR "stress respons*" OR "stress react*" OR "stress exposure*" OR "stress detection*" OR "stress measur*" OR “stress intervention*” OR “stress induc*” OR “Trier Social Stress Test” OR TSST OR “social* threat*” OR “threat* social”)
	OR	Abstract	("acute stress" OR "psycho* stress" OR "stress respons*" OR "stress react*" OR "stress exposure*" OR "stress detection*" OR "stress measur*" OR “stress intervention*” OR “stress induc*” OR “Trier Social Stress Test” OR TSST OR “social* threat*” OR “threat* social”)
Concept 2: Macroscopic body movements	AND	Title	(motion* OR gait OR postur* OR sway OR gesture* OR "Facial Action Coding System" OR "facial expression*" OR “action unit*” OR freezing OR biometrics OR "pressure distribution" OR "capacitive mouse" OR "pressure sensitive")
	OR	Abstract	(motion* OR gait OR postur* OR sway OR gesture* OR "Facial Action Coding System" OR "facial expression*" OR “action unit*” OR freezing OR biometrics OR "pressure distribution" OR "capacitive mouse" OR "pressure sensitive")

The entire search process will be conducted by two reviewers (MK, VR) independently, and any discrepancies in study selection will be resolved through discussion and consensus. Thereby, bias will be minimized and reliability and reproducibility of the search results will be enhanced.

### Screening and selection procedure

All identified records from the databases will be imported into *Rayyan* [70] and duplicates will be removed. Two reviewers (MK, VR) will then screen the titles and abstracts of the remaining records to identify potentially eligible studies. Any disagreements or uncertainties during this stage will be resolved through discussion or, if necessary, by consulting a third reviewer (LA). In the next step, full texts of the selected articles will be retrieved and assessed by the two reviewers against the predetermined eligibility criteria.

### Data collection process

Data of eligible studies will be extracted independently by two authors (MK, VR) and imported into *Excel* [71]. This step will be pre-tested with five articles to ensure feasibility and comprehensiveness. We will contact authors in case of missing data. If there are multiple reports on one set of data, only one report will be included. We will report on the percentage of agreement between the two raters as well as calculate Cohen’s kappa (*K*) to determine the inter-rater reliability.

### Data processing and classification of outcome variables

Extracted data will include information on the title of the paper, author(s), year of publication, country of recruitment, sample size, number of exclusions, reason(s) for exclusion, final participant number, mean age, SD age, percentage of female participants, sample type (general population vs. specific sub-population), inclusion criteria, measure(s) of stress, type of macroscopic movements, description of the stress intervention, description of the control intervention, order of conditions (in case of within-subject design), mean and standard deviation of used stress metric at pre-assessment and post-assessment for the experimental group and control group, and received compensation for participation. Considered studies will be assessed for their methodological quality. We will use appropriate tools, such as the revised Cochrane Risk of Bias tool [72] or ROBINS-I [73] to evaluate the quality of randomized and non-randomized trials, respectively. The assessment will be performed independently by the two reviewers (MK, VR), and any discrepancies will be resolved through consensus or consultation with a third reviewer (LA).

### Data synthesis

First, a qualitative summary of the extracted information from each study and of our risk of bias assessment will be provided in narrative and tabular form. If there is significant variability and unsuitability for statistical pooling and, thus, meta-analytic analyses prove to not be feasible, we will utilize graphical summary methods for synthesizing evidence when a meta-analysis is not feasible. This includes employing harvest plots, effect directions, or bubble plots to summarize information in a manner that is both accessible and user-friendly. Second, if an adequate number of high-quality studies with relatively low heterogeneity are obtained, we will quantitatively synthesize data from primary studies through a meta-analysis. Heterogeneity will be assessed by using *I*^2^ statistics [74] and Cochran’s *Q* test [75]. For those studies that report on both traditional markers and a macroscopic movement, we will compare effect sizes between the two measures. In addition, when correlations between traditional markers and macroscopic movements are reported, we will analyze the correlation coefficients meta-analytically. All analyses will be conducted using Rstudio (version 2023.12.1 + 402 or later). Meta-analyses will be performed using the meta [76] and/or metafor [77] packages, and additional analyses will use relevant packages as appropriate.

### Additional analyses

#### Meta-biases

The results of this review and meta-analyses will undergo critical examination towards meta-biases, such as selective reporting within studies or publication bias across studies. We will construct a funnel plot and conduct tests for asymmetry, such as Egger’s test [78], with a minimum of 10 studies (whenever feasible) to assess the presence of small-study effects [79, 80].

#### Confidence in cumulative evidence

The robustness of the evidence base will be evaluated using the Grading of Recommendations Assessment, Development, and Evaluation (GRADE) system, a framework for appraising the quality of evidence and strength of recommendations [81, 82]. Quality of evidence refers to the certainty that estimated effects are accurate and can be categorized into high, moderate, low, and very low [81, 83]. We will assess the certainty of evidence separately for each primary and secondary outcome. Factors that compromise the quality of results are, among others, study limitations, inconsistent results, indirect evidence, imprecision, and publication bias; if these five factors are present, the quality rating can be downgraded [84]. The strength of the recommendation is characterized by the confidence that the positive effects of an intervention outweigh its negative effects and can be classified as either strong or weak [81, 85]_._

## Discussion

We propose a study protocol for a systematic review and meta-analysis on stress assessment based on body movements and behavior. As a first step, we propose to discriminate between macroscopic movements and microscopic movements. While systematic reviews focusing on internal physiological processes that affect microscopic movements are available (e.g., [86]), findings on macroscopic movements to this day have, to the best of our knowledge, not been assembled in a systematic way. Hence, to offer a more profound insight into this novel approach to stress assessment, and to provide a sound basis for further research, we plan to conduct a systematic review and (if possible) meta-analysis on the effects of acute stress on macroscopic movements, i.e., all body movements that are actively, consciously, or unconsciously initiated by the muscular system. The aim is to determine whether macroscopic movements can be an indicator for acute stress and, if so, which movements best distinguish between a stressful and non-stressful state in human beings. In light of recent technological advances, our findings could further advance unobtrusive and continuous stress detection using tools such as motion sensors, wearable devices, or cameras. This could not only be beneficial in laboratory stress research, but may also be applied in real-world settings, where early interventions or adaptive support are conceivable.

## Supplementary Information

Additional file 1: PRISMA-P guidelines.

## Data Availability

Not applicable.

## Abbreviations

ANS: Autonomic nervous system
AU: Action unit
CNS: Central nervous system
ECG: Electrocardiogram
EEG: Electroencephalogram
GRADE: Grading of Recommendations Assessment, Development, and Evaluation
HPA axis: Hypothalamic–pituitary–adrenal axis
HR: Heart rate
HRV: Heart rate variability
MIST: Montreal Imaging Stress Task
PANAS: Positive Affect Negative Affect Schedule
PASA: Primary Appraisal Secondary Appraisal
PECO: Participants, Exposure, Comparison, Outcomes
PICO: Participants, Intervention, Comparison, Outcomes
SNS: Sympathetic Nervous System
SSSQ: Short Stress State Questionnaire
TSST: Trier Social Stress Test

## References

[1] Sternberg EM. The balance within: the science connecting health and emotions. Macmillan; 2001.

[2] Valentino RJ, Van Bockstaele E. Convergent regulation of locus coeruleus activity as an adaptive response to stress. Eur J Pharmacol. 2008;583:194–203. 18255055 10.1016/j.ejphar.2007.11.062 PMC2349983

[3] Gold PW. The organization of the stress system and its dysregulation in depressive illness. Mol Psychiatry. 2015;20:32–47. 25486982 10.1038/mp.2014.163

[4] Carlson NR. Physiologische psychologie. München: Pearson Studium; 2004.

[5] Rohleder N, Beulen SE, Chen E, Wolf JM, Kirschbaum C. Stress on the dance floor: the cortisol stress response to social-evaluative threat in competitive ballroom dancers. Pers Soc Psychol Bull. 2007;33:69–84. 17178931 10.1177/0146167206293986

[6] Smeets T, Cornelisse S, Quaedflieg CWEM, Meyer T, Jelicic M, Merckelbach H. Introducing the Maastricht Acute Stress Test (MAST): a quick and non-invasive approach to elicit robust autonomic and glucocorticoid stress responses. Psychoneuroendocrinology. 2012;37:1998–2008. 22608857 10.1016/j.psyneuen.2012.04.012

[7] Gjerstad JK, Lightman SL, Spiga F. Role of glucocorticoid negative feedback in the regulation of HPA axis pulsatility. Stress. 2018;21:403–16. 29764284 10.1080/10253890.2018.1470238 PMC6220752

[8] Sapolsky RM, Romero LM, Munck AU. How do glucocorticoids influence stress responses? Integrating permissive, suppressive, stimulatory, and preparative actions. Endocr Rev. 2000;21:55–89. 10696570 10.1210/edrv.21.1.0389

[9] Chen X, Gianferante D, Hanlin L, Fiksdal A, Breines JG, Thoma MV, et al. HPA-axis and inflammatory reactivity to acute stress is related with basal HPA-axis activity. Psychoneuroendocrinology. 2017;78:168–76. 28209543 10.1016/j.psyneuen.2017.01.035 PMC5375039

[10] Allen AP, Kennedy PJ, Dockray S, Cryan JF, Dinan TG, Clarke G. The trier social stress test: principles and practice. Neurobiol Stress. 2017;6:113–26. 28229114 10.1016/j.ynstr.2016.11.001 PMC5314443

[11] Rohleder N. Stress and inflammation - the need to address the gap in the transition between acute and chronic stress effects. Psychoneuroendocrinology. 2019;105:164–71. 30826163 10.1016/j.psyneuen.2019.02.021

[12] Dhabhar FS. Effects of stress on immune function: the good, the bad, and the beautiful. Immunol Res. 2014;58:193–210. 24798553 10.1007/s12026-014-8517-0

[13] Herman JP, McKlveen JM, Ghosal S, Kopp B, Wulsin A, Makinson R, et al. Regulation of the hypothalamic-pituitary-adrenocortical stress response. Compr Physiol. 2016;6:603–21. 27065163 10.1002/cphy.c150015 PMC4867107

[14] McEwen BS. Physiology and neurobiology of stress and adaptation: central role of the brain. Physiol Rev. 2007;87:873–904. 17615391 10.1152/physrev.00041.2006

[15] Ulrich-Lai YM, Herman JP. Neural regulation of endocrine and autonomic stress responses. Nat Rev Neurosci. 2009;10:397–409. 19469025 10.1038/nrn2647 PMC4240627

[16] Lazarus RS, Speisman JC, Mordkoff AM, Davison LA. A laboratory study of psychological stress produced by a motion picture film. Psychological Monographs: General and Applied. 1962;76:1.

[17] Lehman BJ, Kirsch JA, Jones DR. Effectively analyzing change over time in laboratory research on stress and health: a multilevel modeling approach. Soc Personal Psychol Compass. 2015;9:551–66.

[18] Bali A, Jaggi AS. Clinical experimental stress studies: methods and assessment. Rev Neurosci. 2015;26:555–79. 26020552 10.1515/revneuro-2015-0004

[19] Schlotz W. Investigating associations between momentary stress and cortisol in daily life: what have we learned so far? Psychoneuroendocrinology. 2019;105:105–16. 30503527 10.1016/j.psyneuen.2018.11.038

[20] Schwabe L, Haddad L, Schachinger H. HPA axis activation by a socially evaluated cold-pressor test. Psychoneuroendocrinology. 2008;33:890–5. 18403130 10.1016/j.psyneuen.2008.03.001

[21] Dedovic K, Renwick R, Mahani NK, Engert V, Lupien SJ, Pruessner JC. The Montreal imaging stress task: using functional imaging to investigate the effects of perceiving and processing psychosocial stress in the human brain. J Psychiatry Neurosci. 2005;30:319–25. 16151536 PMC1197276

[22] Kirschbaum C, Pirke K-M, Hellhammer DH. The ‘Trier Social Stress Test’–a tool for investigating psychobiological stress responses in a laboratory setting. Neuropsychobiology. 1993;28:76–81. 8255414 10.1159/000119004

[23] Dickerson SS, Kemeny ME. Acute stressors and cortisol responses: a theoretical integration and synthesis of laboratory research. Psychol Bull. 2004;130:355–91. 15122924 10.1037/0033-2909.130.3.355

[24] Helton WS. Validation of a short stress state questionnaire. Proceedings of the Human Factors and Ergonomics Society Annual Meeting. 2004;48:1238–42.

[25] Ringgold V, Shields GS, Hauck F, Kurz M, Schindler-Gmelch L, Abel L, et al. The Short Stress State Questionnaire in German (SSSQ-G): A multi-study validation. Eur J Health Psychol. 2024;31(4):189–200.

[26] Watson D, Clark LA, Tellegen A. Development and validation of brief measures of positive and negative affect: the PANAS scales. J Pers Soc Psychol. 1988;54:1063–70. 3397865 10.1037//0022-3514.54.6.1063

[27] Gaab J. PASA – Primary appraisal secondary appraisal. Verhaltenstherapie. 2009;19:114–5.

[28] Alberdi A, Aztiria A, Basarab A. Towards an automatic early stress recognition system for office environments based on multimodal measurements: a review. J Biomed Inform. 2016;59:49–75. 26621099 10.1016/j.jbi.2015.11.007

[29] Campbell J, Ehlert U. Acute psychosocial stress: does the emotional stress response correspond with physiological responses? Psychoneuroendocrinology. 2012;37:1111–34. 22260938 10.1016/j.psyneuen.2011.12.010

[30] Kirschbaum C, Hellhammer DH. Salivary cortisol in psychoneuroendocrine research: recent developments and applications. Psychoneuroendocrinology. 1994;19:313–33. 8047637 10.1016/0306-4530(94)90013-2

[31] Labuschagne I, Grace C, Rendell P, Terrett G, Heinrichs M. An introductory guide to conducting the Trier Social Stress Test. Neurosci Biobehav Rev. 2019;107:686–95. 31560923 10.1016/j.neubiorev.2019.09.032

[32] Nater UM, Rohleder N. Salivary alpha-amylase as a non-invasive biomarker for the sympathetic nervous system: current state of research. Psychoneuroendocrinology. 2009;34:486–96. 19249160 10.1016/j.psyneuen.2009.01.014

[33] Levine A, Zagoory-Sharon O, Feldman R, Lewis JG, Weller A. Measuring cortisol in human psychobiological studies. Physiol Behav. 2007;90:43–53. 17055006 10.1016/j.physbeh.2006.08.025

[34] Tvarijonaviciute A, Martinez-Lozano N, Rios R, de Marcilla Teruel MC, Garaulet M, Cerón JJ. Saliva as a non-invasive tool for assessment of metabolic and inflammatory biomarkers in children. Clin Nutr. 2020;39:2471–8. 31787367 10.1016/j.clnu.2019.10.034

[35] Allen AP, Kennedy PJ, Cryan JF, Dinan TG, Clarke G. Biological and psychological markers of stress in humans: focus on the Trier Social Stress Test. Neurosci Biobehav Rev. 2014;38:94–124. 24239854 10.1016/j.neubiorev.2013.11.005

[36] Fries E, Dettenborn L, Kirschbaum C. The cortisol awakening response (CAR): facts and future directions. Int J Psychophysiol. 2009;72:67–73. 18854200 10.1016/j.ijpsycho.2008.03.014

[37] Hansen AM, Garde AH, Persson R. Sources of biological and methodological variation in salivary cortisol and their impact on measurement among healthy adults: a review. Scand J Clin Lab Invest. 2008;68:448–58. 18609093 10.1080/00365510701819127

[38] Burke HM, Davis MC, Otte C, Mohr DC. Depression and cortisol responses to psychological stress: a meta-analysis. Psychoneuroendocrinology. 2005;30:846–56. 15961250 10.1016/j.psyneuen.2005.02.010

[39] Zorn JV, Schür RR, Boks MP, Kahn RS, Joëls M, Vinkers CH. Cortisol stress reactivity across psychiatric disorders: a systematic review and meta-analysis. Psychoneuroendocrinology. 2017;77:25–36. 28012291 10.1016/j.psyneuen.2016.11.036

[40] Herhaus B, Petrowski K. Cortisol stress reactivity to the Trier Social Stress Test in obese adults. Obes Facts. 2018;11:491–500. 30537716 10.1159/000493533 PMC6341320

[41] Gradl S, Wirth M, Richer R, Rohleder N, Eskofier BM. An Overview of the Feasibility of Permanent, Real-Time, Unobtrusive Stress Measurement with Current Wearables. In: Proceedings of the 13th EAI International Conference on Pervasive Computing Technologies for Healthcare. Association for Computing Machinery, New York, NY, USA, 2019. pp. 360–365.

[42] Inder WJ, Dimeski G, Russell A. Measurement of salivary cortisol in 2012 - laboratory techniques and clinical indications. Clin Endocrinol. 2012;77:645–51. 10.1111/j.1365-2265.2012.04508.x 22812714

[43] Janson J, Rohleder N. Distraction coping predicts better cortisol recovery after acute psychosocial stress. Biol Psychol. 2017;128:117–24. 28743456 10.1016/j.biopsycho.2017.07.014

[44] Kim H-G, Cheon E-J, Bai D-S, Lee YH, Koo B-H. Stress and heart rate variability: a meta-analysis and review of the literature. Psychiatry Investig. 2018;15:235–45. 29486547 10.30773/pi.2017.08.17 PMC5900369

[45] Dalmeida KM, Masala GL. HRV features as viable physiological markers for stress detection using wearable devices. Sensors. 2021. 10.3390/s21082873. 33921884 10.3390/s21082873 PMC8072791

[46] Pourmohammadi S, Maleki A. Stress detection using ECG and EMG signals: a comprehensive study. Comput Methods Programs Biomed. 2020;193:105482. 32408236 10.1016/j.cmpb.2020.105482

[47] Rieger A, Stoll R, Kreuzfeld S, Behrens K, Weippert M. Heart rate and heart rate variability as indirect markers of surgeons’ intraoperative stress. Int Arch Occup Environ Health. 2014;87:165–74. 23370764 10.1007/s00420-013-0847-z

[48] Schiweck C, Piette D, Berckmans D, Claes S, Vrieze E. Heart rate and high frequency heart rate variability during stress as biomarker for clinical depression. A systematic review. Psychol Med. 2019;49:200–11. 30134999 10.1017/S0033291718001988

[49] Zhang Y, Weaver RG, Armstrong B, Burkart S, Zhang S, Beets MW. Validity of wrist-worn photoplethysmography devices to measure heart rate: a systematic review and meta-analysis. J Sports Sci. 2020;38:2021–34. 32552580 10.1080/02640414.2020.1767348

[50] Rosenberg EL, Ekman P. What the face reveals: basic and applied studies of spontaneous expression using the Facial Action Coding System (FACS). Oxford University Press; 2020.

[51] Ekman P, Friesen WV. Constants across cultures in the face and emotion. J Pers Soc Psychol. 1971;17:124–9. 5542557 10.1037/h0030377

[52] Lupis SB, Lerman M, Wolf JM. Anger responses to psychosocial stress predict heart rate and cortisol stress responses in men but not women. Psychoneuroendocrinology. 2014;49:84–95. 25064831 10.1016/j.psyneuen.2014.07.004 PMC4165699

[53] Blasberg JU, Gallistl M, Degering M, Baierlein F, Engert V. You look stressed: a pilot study on facial action unit activity in the context of psychosocial stress. Comprehensive Psychoneuroendocrinology. 2023;15:100187. 37577295 10.1016/j.cpnec.2023.100187 PMC10422124

[54] Velusamy S, Kannan H, Anand B, Sharma A, Navathe B. A method to infer emotions from facial Action Units. In: 2011 IEEE International Conference on Acoustics, Speech and Signal Processing (ICASSP). ieeexplore.ieee.org, 2011. pp. 2028–2031.

[55] Giannakakis G, Pediaditis M, Manousos D, Kazantzaki E, Chiarugi F, Simos PG, et al. Stress and anxiety detection using facial cues from videos. Biomed Signal Process Control. 2017;31:89–101.

[56] Roelofs K, Hagenaars MA, Stins J. Facing freeze: social threat induces bodily freeze in humans. Psychol Sci. 2010;21:1575–81. 20876881 10.1177/0956797610384746

[57] Hagenaars MA, Roelofs K, Stins JF. Human freezing in response to affective films. Anxiety Stress Coping. 2014;27:27–37. 23805855 10.1080/10615806.2013.809420

[58] Richer R, Koch V, Abel L, et al. Machine learning-based detection of acute psychosocial stress from body posture and movements. Sci Rep. 2024;14:8251. 38589504 10.1038/s41598-024-59043-1 PMC11375162

[59] Pisanski K, Kobylarek A, Jakubowska L, et al. Multimodal stress detection: testing for covariation in vocal, hormonal and physiological responses to Trier Social Stress Test. Horm Behav. 2018;106:52–61. 30189213 10.1016/j.yhbeh.2018.08.014

[60] Arnrich B, Setz C, La Marca R, Tröster G, Ehlert U. What does your chair know about your stress level? IEEE Trans Inf Technol Biomed. 2010;14:207–14. 19887325 10.1109/TITB.2009.2035498

[61] Paredes PE, Ordonez F, Ju W, Landay JA. Fast & Furious: Detecting Stress with a Car Steering Wheel. In: Proceedings of the 2018 CHI Conference on Human Factors in Computing Systems. Association for Computing Machinery, New York, NY, USA, 2018. pp 1–12.

[62] Lim YM, Ayesh A, Stacey M. Detecting cognitive stress from keyboard and mouse dynamics during mental arithmetic. In: 2014 Science and Information Conference. ieeexplore.ieee.org, 2014. pp 146–152.

[63] Vizer LM, Zhou L, Sears A. Automated stress detection using keystroke and linguistic features: an exploratory study. Int J Hum Comput Stud. 2009;67:870–86.

[64] Freihaut P, Göritz AS. Does peoples’ keyboard typing reflect their stress level? Z Psychol. 2021;229:245–50.

[65] Wetherell MA, Lau S-H, Maxion RA. The effect of socially evaluated multitasking stress on typing rhythms. Psychophysiology. 2023. 10.1111/psyp.14293. 36938968 10.1111/psyp.14293

[66] Liu W, Bao Q, Sun Y, Mei T. Recent advances of monocular 2D and 3D human pose estimation: a deep learning perspective. ACM Comput Surv. 2022;55:1–41.

[67] Moher D, Shamseer L, Clarke M, Ghersi D, Liberati A, Petticrew M, Shekelle P, Stewart LA, PRISMA-P Group. Preferred reporting items for systematic review and meta-analysis protocols (PRISMA-P) 2015 statement. Syst Rev. 2015;4:1. 25554246 10.1186/2046-4053-4-1 PMC4320440

[68] Richardson WS, Wilson MC, Nishikawa J, Hayward RS. The well-built clinical question: a key to evidence-based decisions. ACP J Club. 1995;123:A12–3. 7582737 10.7326/ACPJC-1995-123-3-A12

[69] Godin K, Stapleton J, Kirkpatrick SI, Hanning RM, Leatherdale ST. Applying systematic review search methods to the grey literature: a case study examining guidelines for school-based breakfast programs in Canada. Syst Rev. 2015;4:138. 26494010 10.1186/s13643-015-0125-0 PMC4619264

[70] Ouzzani M, Hammady H, Fedorowicz Z, Elmagarmid A. Rayyan-a web and mobile app for systematic reviews. Syst Rev. 2016;5:210. 27919275 10.1186/s13643-016-0384-4 PMC5139140

[71] Microsoft Corporation. Microsoft Excel. 2023.

[72] Sterne JA, Savović J, Page MJ, et al. RoB 2: a revised tool for assessing risk of bias in randomised trials. BMJ. 2019;366:l4898. 31462531 10.1136/bmj.l4898

[73] Sterne JA, Hernán MA, Reeves BC, et al. ROBINS-I: a tool for assessing risk of bias in non-randomised studies of interventions. BMJ. 2016;355:i4919. 27733354 10.1136/bmj.i4919 PMC5062054

[74] Higgins JPT, Thompson SG, Deeks JJ, Altman DG. Measuring inconsistency in meta-analyses. BMJ. 2003;327:557–60. 12958120 10.1136/bmj.327.7414.557 PMC192859

[75] Cochran WG. The combination of estimates from different experiments. Biometrics. 1954;10:101–29.

[76] Schwarzer G, Carpenter JR, Rucker G. Meta-Analysis with R. 1st ed. 2015. 10.1007/978-3-319-21416-0.

[77] Viechtbauer W (2010) Conducting meta-analyses inRwith themetaforPackage. J Stat Softw. 10.18637/jss.v036.i03

[78] Egger M, Davey Smith G, Schneider M, Minder C. Bias in meta-analysis detected by a simple, graphical test. BMJ. 1997;315:629–34. 9310563 10.1136/bmj.315.7109.629 PMC2127453

[79] Sterne JA, Gavaghan D, Egger M. Publication and related bias in meta-analysis: power of statistical tests and prevalence in the literature. J Clin Epidemiol. 2000;53:1119–29. 11106885 10.1016/s0895-4356(00)00242-0

[80] Sterne JAC, Sutton AJ, Ioannidis JPA, et al. Recommendations for examining and interpreting funnel plot asymmetry in meta-analyses of randomised controlled trials. BMJ. 2011;343:d4002. 21784880 10.1136/bmj.d4002

[81] Guyatt GH, Oxman AD, Vist GE, Kunz R, Falck-Ytter Y, Alonso-Coello P, Schünemann HJ, GRADE Working Group. Grade: an emerging consensus on rating quality of evidence and strength of recommendations. BMJ. 2008;336:924–6. 18436948 10.1136/bmj.39489.470347.AD PMC2335261

[82] Atkins D, Best D, Briss PA, et al. Grading quality of evidence and strength of recommendations. BMJ. 2004;328:1490. 15205295 10.1136/bmj.328.7454.1490 PMC428525

[83] Balshem H, Helfand M, Schünemann HJ, et al. Grade guidelines: 3. Rating the quality of evidence. J Clin Epidemiol. 2011;64:401–6. 21208779 10.1016/j.jclinepi.2010.07.015

[84] Guyatt GH, Oxman AD, Kunz R, Vist GE, Falck-Ytter Y, Schünemann HJ, GRADE Working Group. What is “quality of evidence” and why is it important to clinicians? BMJ. 2008;336:995–8. 18456631 10.1136/bmj.39490.551019.BE PMC2364804

[85] Andrews J, Guyatt G, Oxman AD, et al. Grade guidelines: 14. Going from evidence to recommendations: the significance and presentation of recommendations. J Clin Epidemiol. 2013;66:719–25. 23312392 10.1016/j.jclinepi.2012.03.013

[86] Castaldo R, Melillo P, Bracale U, Caserta M, Triassi M, Pecchia L. Acute mental stress assessment via short term HRV analysis in healthy adults: a systematic review with meta-analysis. Biomed Signal Process Control. 2015;18:370–7.

